# Breast Abscess Caused by Salmonella paratyphi A in an Immunocompetent Non-Lactating Female: A Case Report

**DOI:** 10.12669/pjms.42.(11AASC).15805

**Published:** 2026-04

**Authors:** Hafiz Syed Muhammad Mubashir, Faaiz Rasheed, Soobia Rehman

**Affiliations:** 1 Hafiz Syed Muhammad Mubashir, MBBS. Department of General Surgery, Memon Medical Institute Hospital, Karachi, Pakistan; 2 Faaiz Rasheed, MBBS. Department of General Surgery, Memon Medical Institute Hospital, Karachi, Pakistan; 3 Soobia Rehman, MBBS, FCPS. Department of General Surgery, Memon Medical Institute Hospital, Karachi, Pakistan

**Keywords:** Breast abscess, Extra-intestinal infection, Non-lactating female, *Salmonella enterica Paratyphi A*

## Abstract

**Background:**

Breast abscesses are most commonly caused by Staphylococcus aureus, whereas *Salmonella enterica Paratyphi A* is an exceptionally rare pathogen, even in regions where enteric fever is endemic. Extra-intestinal infection due to *Salmonella enterica Paratyphi A* is uncommon, and involvement of the breast is particularly rare.

**Case Presentation:**

We report a case of a 36-year-old immunocompetent, non-lactating female who presented with a four week history of pain and swelling in the right breast. Ultrasound revealed a localized abscess, which was aspirated yielding purulent material. Culture identified *Salmonella enterica* serovar *Paratyphi A*, sensitive to ampicillin and ceftriaxone but resistant to ciprofloxacin. The patient responded completely to targeted antibiotic therapy following drainage, with no recurrence on follow-up.

**Conclusion:**

This case highlights an uncommon presentation of *S. paratyphi A* in a healthy individual without systemic features of enteric fever. Awareness of this rare entity is essential, as clinical features may mimic pyogenic or tubercular mastitis, leading to delayed or inappropriate therapy. Culture confirmation and antibiotic susceptibility testing remain crucial for optimal management and favorable outcomes.

## INTRODUCTION

Breast abscess is a localized collection of pus within breast tissue, commonly occurring as a complication of lactational mastitis. *Staphylococcus aureus* remains the primary pathogen, followed by streptococci and anaerobes.^1^,^2^ Conversely, breast infection by *Salmonella* species is exceedingly rare, even in areas where enteric fever is endemic. *Salmonella enterica* includes numerous serovars responsible for typhoidal and non-typhoidal infections; while *S. typhi* and *S. paratyphi* typically cause systemic febrile illness, extra-intestinal localization such as abscess formation is uncommon and usually follows transient or silent bacteremia.^1^,^3^

Reports of *Salmonella enterica Paratyphi A* causing breast abscess are exceedingly rare, with only a few recorded cases worldwide.^1^,^4^ The majority of instances have been observed in Asian populations, predominantly among non-lactating women without systemic features of enteric fever. Proposed mechanisms include hematogenous seeding from transient bacteremia, reactivation from a carrier state, or direct translocation from the gastrointestinal tract.^3^,^5^ Immunosuppressive conditions such as diabetes or steroid use predispose individuals to such infection,^2^,^6^ Several cases involving healthy, immunocompetent individuals have also been reported.^1^,^7^,^8^

Clinically, these abscesses often resemble pyogenic or tubercular mastitis, leading to delayed or inappropriate treatment.^4^,^5^,^9^ Empirical antibiotics may be ineffective in recurrent or chronic infections, highlighting the importance of microbiological confirmation and targeted therapy.^5^,^6^ Most isolates remain susceptible to ampicillin and ceftriaxone, nevertheless, the rising resistance to fluoroquinolone underscores the necessity for susceptibility testing.^1^

## CASE PRESENTATION

A 36 years old female with no known comorbidities presented to the surgical outpatient department with complaints of pain and swelling in the right breast for approximately four weeks. The swelling had gradually increased in size over the past month. She denied fever, nipple discharge, trauma, or cyclic variation in symptoms. The patient was married, had one child delivered via cesarean section four years earlier, and had breastfed for six months. Her menstrual cycles were regular and she did not use hormonal contraceptives. There was no family history of malignancy or tuberculosis.

On examination, a 4×4 cm tender lump was palpable in the retro-areolar region at the 4o’clock position of the right breast. The overlying skin was normal with no erythema, induration, or nipple retraction. The contralateral breast and axillae were normal, with no palpable lymphadenopathy.

**Fig.1:**
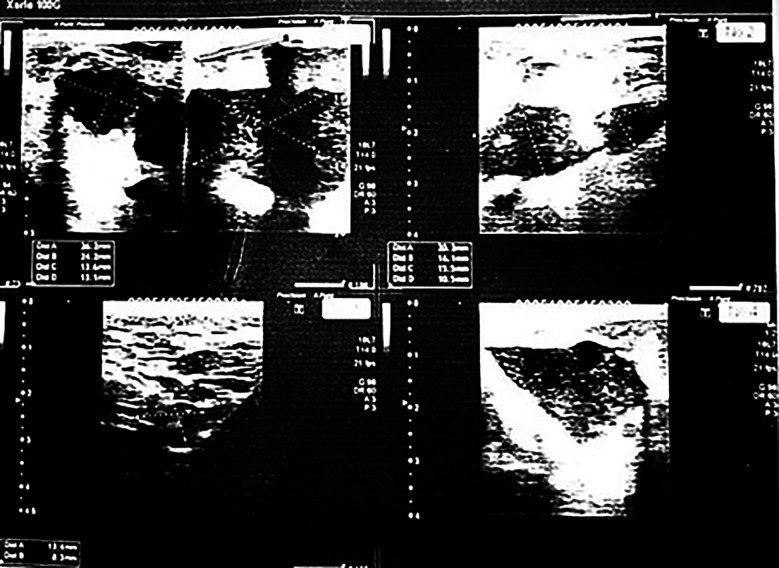
Ultrasound of the right breast demonstrating a well-defined hypoechoic lesion with internal echoes in the retro-areolar region, consistent with a localized abscess cavity.

Ultrasound of the right breast revealed a well-circumscribed hypoechoic lesion consistent with an abscess cavity (Fig.1). Approximately 10ml of thick, purulent material was aspirated and sent for microbiological analysis. Empirical oral co-amoxiclav (1 g twice daily) was initiated for five days.

Initial laboratory investigations showed a normal complete blood count (Hb 13.1 g/dL, TLC 9.5 × 10^9^/L, neutrophils 63%) and ESR 23 mm/h. HbA1c was 5.94%, ruling out diabetes. Ziehl–Neelsen staining of the pus was negative for acid-fast bacilli, and GeneXpert MTB/RIF assay was also negative for *Mycobacterium tuberculosis* DNA. Gram stain of the aspirated pus demonstrated numerous polymorphonuclear leukocytes and a few gram-negative bacilli.

On the subsequent visit another aspiration was performed draining 5ml of purulent discharge. Aerobic culture yielded non-lactose-fermenting colonies identified as *Salmonella enterica* serovar Paratyphi A. The isolate was sensitive to ampicillin, ceftriaxone, and co-trimoxazole, but resistant to ciprofloxacin. No anaerobes were isolated. Based on the susceptibility pattern, the antibiotic regimen was changed to oral ampicillin–cloxacillin (500 mg four times daily) for seven days.

At follow-up one week later, the patient reported marked symptomatic improvement, and the swelling had significantly decreased. A repeat ultrasound performed after four weeks demonstrated complete resolution with no residual cavity or recurrence.

## DISCUSSION

Breast abscesses are frequent infections, particularly among lactating women, with Staphylococcus aureus being the prominent causative agent.^1^,^2^ In contrast, abscesses induced by Salmonella species are exceptionally uncommon, even in endemic regions. The first case was documented by Thayer and Hazen in 1907, and subsequently, only a limited number of S. paratyphi A breast abscesses have been reported worldwide.^10^ Notably, the majority of reported cases have been observed in Asian populations, indicating potential regional influences or exposure patterns.

*Salmonella enterica Paratyphi A* is a human-restricted serovar that typically causes paratyphoid fever. Extra-intestinal localization, particularly in the breast, is exceedingly rare and is believed to arise through hematogenous dissemination during transient or silent bacteremia.^1^,^3^,^5^ Alternative pathways include reactivation from a carrier state or direct invasion from the gastrointestinal tract.^3^,^8^

Although Salmonella breast abscesses have been reported in immunocompromised patients,^2^,^6^ numerous cases, including the present one, have been seen in immunocompetent women.^1^,^7^ The patient’s, normal HbA1c and negative blood cultures suggest a localized infection secondary to transient bacteremia. However, HIV testing was not performed in this patient, which represents a limitation of this report, given the known association between invasive *Salmonella* infections and HIV infection.

Clinically, these abscesses may resemble pyogenic or tuberculous infections, making diagnosis challenging. Painful swelling, localized tenderness, and intermittent purulent discharge are characteristics, whereas systemic features such as fever are rare.^2^,^4^,^5^ The overlap results in frequent misdiagnosis and potential failure of empirical therapy. Fernando et al. (2012) and Ghadage et al. (2014) reported treatment failure with oral azithromycin and broad-spectrum antibiotics until *Salmonella* was identified and organism-specific therapy commenced.^4^,^5^ In our instance, a partial response to co-amoxiclav followed by complete resolution after targeted ampicillin therapy reflects these findings and underscores the necessity of microbiological confirmation.

Most isolates continue to exhibit sensitivity to ampicillin, ceftriaxone, and co-trimoxazole but demonstrating increasing resistance to fluoroquinolone.^1^,^9^ Drainage, along with appropriate antimicrobial therapy ensures recovery, as evidenced by previous publications.^1^,^2^,^7^ The absence of systemic bacteremia or recurrence substantiates transient bacteremia as a plausible mechanism.

Therapeutic efficacy depends on both drainage and targeted antibiotics. In the review by Agrawal et al., all patients who underwent drainage with appropriate antimicrobial therapy achieved complete recovery.^1^ Similar results were observed in the reports of Deshpande et al. and Jain et al.; our patient also experienced full clinical and radiologic resolution after aspiration and a short course of organism-specific treatment.^2^,^6^

Interestingly, blood and stool cultures are often negative, suggesting that localized abscess formation can occur without systemic bacteremia.^1^,^5^ This supports the hypothesis of transient bacteremia or localized inoculation as plausible mechanisms. The absence of recurrence on follow-up further indicates that early identification and targeted therapy are key to favorable outcomes.

This case therefore expands the limited global experience with *S. paratyphi A* breast abscess in immunocompetent, non-lactating females. Clinicians should maintain suspicion for atypical organisms when conventional therapy fails, or gram-negative bacilli are observed on smear. Routine culture and susceptibility testing of aspirated pus are essential for accurate diagnosis and successful management.

## CONCLUSION

*Salmonella enterica Paratyphi A* breast abscess is an uncommon yet treatable condition that may arise in healthy, non-lactating women. Due to its appearance resembling common bacterial abscesses, culture confirmation is essential for diagnosis. Combined drainage and targeted antimicrobial therapy lead to excellent outcomes. Recognizing this unusual presentation is essential for prompt recognition and prevention of recurrence.

### Author`s Contributions:

**FR:** Conception of the study and data collection.

**HSMM:** Manuscript writing, data analysis, and literature review.

**SR:** Critical review and final approval of the manuscript.

All authors have read and approved the final manuscript and are responsible and accountable for the accuracy and integrity of the work.
